# Overall coronary disease burden modifies the prognostic benefit of CTO-PCI: a SYNTAX score–stratified meta-analysis

**DOI:** 10.1093/ehjopen/oeag045

**Published:** 2026-03-12

**Authors:** Nino Cocco, Kambis Mashayekhi, Agostino Spanò, Michael Behnes, Pierfrancesco Agostoni, Daniel Weilenmann, Claudiu Ungureanu, Giuseppe Colletti, Maourane Boukhris, Giulio Cocco, Cammalleri Valeria, Annunziata Nusca, Gian Paolo Ussia, Gregor Leibundgut

**Affiliations:** Department of Cardiovascular Sciences, Campus Bio-Medico University of Rome, Via Álvaro del Portillo 21, Rome 00128, Italy; Heart Center Lahr, Internal Medicine and Cardiology, Hohbergweg, Lahr 77933, Germany; Department of Cardiovascular Sciences, Campus Bio-Medico University of Rome, Via Álvaro del Portillo 21, Rome 00128, Italy; First Department of Medicine, University Hospital Mannheim, Faculty of Medicine Mannheim, Heidelberg University, Theodor-Kutzer-Ufer 1-3, Mannheim 68167, Germany; HartCentrum, Ziekenhuis Netwerk Antwerpen (ZNA), Middelheim Lindendreef 1, Antwerp 2020, Belgium; Cardiology, Cantonal Hospital St Gallen, Rorschacher Strasse 95, St Gallen 9007, Switzerland; Department of Cardiology, Hôpital de Jolimont, Rue Ferrer 159, La Louvrière 7100, Belgium; Department of Cardiology, Hôpital d‘Arlon, Arlon, Rue des Déportés 137, Arlon 6700, Belgium; Cardiology Department, CHU Saint-Etienne, Avenue Albert Raimond 42055, Saint Étienne 42270, France; Department of Medicine and Aging Sciences, University of Chieti G D'Annunzio, Via Vestini 31, Chieti 66100, Italy; Department of Cardiovascular Sciences, Campus Bio-Medico University of Rome, Via Álvaro del Portillo 21, Rome 00128, Italy; Department of Cardiovascular Sciences, Campus Bio-Medico University of Rome, Via Álvaro del Portillo 21, Rome 00128, Italy; Department of Cardiovascular Sciences, Campus Bio-Medico University of Rome, Via Álvaro del Portillo 21, Rome 00128, Italy; University Heart Center, University Hospital Basel, Petersgraben 4, Basel 4053, Switzerland

**Keywords:** Percutaneous coronary intervention, Chronic total occlusion, Global atherosclerotic burden, Refractory cardiac arrest, Cardiac sudden death

## Abstract

**Aims:**

Extensive coronary artery disease (CAD) coexisting with chronic total occlusion (CTO) is associated with adverse outcomes, yet patients with advanced CAD are often underrepresented in randomized trials, and the prognostic impact of CTO percutaneous coronary intervention (CTO-PCI) across different levels of anatomical complexity remains uncertain. We aimed to determine whether the overall CAD burden, quantified by the SYNTAX score (SS), influences the prognostic effect of CTO-PCI.

**Methods and results:**

A systematic search of PubMed, Embase, Google Scholar, and Cochrane databases was conducted. Eligible studies compared successful CTO-PCI vs. no CTO-PCI and reported the mean SYNTAX score of the cohort. Two reviewers independently extracted data. The primary endpoint was annualized cardiovascular (CV) mortality. Pooled hazard ratios (HRs) were calculated using fixed- or random-effects models with inverse-variance weighting. Meta-regression explored the relationship between SS and CV mortality, and subgroup analyses were performed according to predefined SS categories. Seventeen studies (3 randomized and 14 prospective observational; n = 11 001) were included. Successful CTO-PCI was associated with significantly lower CV mortality compared with non-revascularization (HR 0.54; 95% CI 0.46–0.64; *P* < 0.001). The prognostic benefit increased with CAD complexity, with HRs of 0.61, 0.44, and 0.10 across low (SS < 22), intermediate (SS 23–32), and high (SS > 33) strata, respectively (*P*-trend = 0.04). Meta-regression confirmed a CAD complexity-dependent effect (∼1.5% lower annual CV mortality per 10-point SS increase; *P* = 0.001). These findings apply to a PCI-selected population, as CABG-treated patients were not included.

**Conclusion:**

The survival benefit of CTO-PCI appears to increase with the extent of overall coronary disease, suggesting that patients with higher anatomical burden may derive greater prognostic benefit from successful CTO revascularization.

## Graphical Abstract

**Graphical Abstract:**
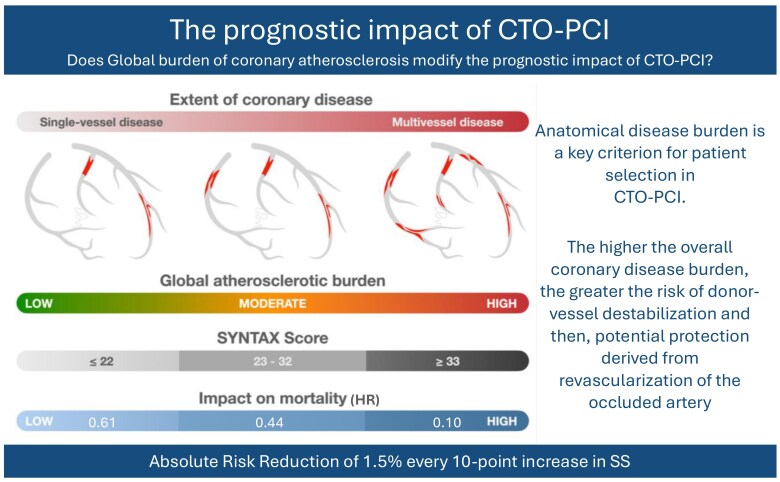

## Introduction

Sudden cardiac death (SCD) remains a major public health concern in coronary artery disease (CAD), with out-of-hospital cardiac arrest (OHCA) being its most frequent presentation.^1^ Despite advances in prevention and acute management, the incidence of SCD and OHCA has remained largely unchanged across different healthcare systems over time,^2^ representing a major clinical challenge, with growing evidence pointing to the prognostic importance of extensive CAD, particularly in the presence of chronic total occlusions (CTOs).^3,4^

It is well established that acute destabilization occurring on the background of chronic CAD frequently underlies cardiac arrest (CA).^5^ Prognosis is particularly unfavourable when the culprit lesion during CA involves a donor vessel supplying a CTO, with such patients facing substantially higher mortality risk,^6^ explaining the increased risk of sudden death associated with CTOs and complex multivessel disease.^3,5^ While coronary revascularization offers clear survival benefit in acute coronary syndromes (ACS), its impact in chronic disease settings remains less consistent, especially in patients with CTO.^7^ Nonetheless, recent data have confirmed the role of chronic coronary disease and CTO in sudden death.^6,8^

Although observational studies suggest a benefit from preventive CTO revascularization,^9^ recent randomized controlled trials (RCTs) have failed to demonstrate consistent improvement in outcomes.^7^ As a result, the optimal management of CTO in the context of multivessel disease (MVD) remains uncertain^10,11^

Traditional revascularization decision-making has relied heavily on ischaemia and viability testing^12–14^ However, accumulating evidence suggests these parameters are limited in guiding treatment selection for revascularization, while overall atherosclerotic burden, rather than the presence or extent of inducible ischaemia, is the dominant determinant of prognosis^7,15–18^ Consistently, both the completeness of revascularization in MVD and the severity of non-occluded coronary artery disease have been identified as key drivers of mortality risk.^16,19,20^ These findings are aligned with observations from Yannopoulos *et al*.^4^ and Kosmopoulos *et al*.^3,^ which emphasize that an extensive atherosclerotic burden substantially increases the likelihood of refractory cardiac arrest, the terminal event in many patients with CTO^8,21–25^

Based on these insights, we hypothesize that the prognostic effect of CTO-PCI is strongly modified by the overall atherosclerotic burden. Thus, patient selection for CTO-PCI should not focus exclusively on the occluded vessel but rather on the extent and severity of concomitant non-CTO disease. In patients with MVD and diffuse atherosclerosis, successful CTO recanalisation may confer a meaningful survival benefit, even if other lesions remain untreated. However, the patients with advanced CAD, particularly those with CTO, are often excluded from randomized controlled trials, and the prognostic impact of CTO percutaneous coronary intervention (CTO-PCI) across varying degrees of CAD complexity remains uncertain.^26^

The SYNTAX score (SS) remains the only standardized tool to quantify epicardial coronary artery disease complexity and overall burden.^11^ Therefore, the present meta-analysis aims to evaluate the impact of CTO-PCI on mortality, stratified by SS, in comparison with untreated CTO-PCI.

## Methods

All data for the analyses included in this study are available within the paper and the Supplementary material online. Additional data not presented are available from the corresponding author upon reasonable request.

The present study was performed according to the Preferred Reporting Items for Systematic Reviews and Meta-Analyses (PRISMA) 2020 guidelines (see Supplementary material online, *Table S1*). The study was designed and registered in the International Prospective Register of Systematic Reviews (PROSPERO, registration number: CRD42023477306).

### Data sources, search strategies, and data collection process

We searched PubMed, EMBASE, MEDLINE, Google Scholar and Cochrane databases Systematic Reviews until April 2024, using the following search terms separately and in combination: CTO, chronic occluded coronary, chronic occluded coronary arteries, percutaneous coronary intervention, coronary angioplasty, mortality, all-cause death, cardiovascular death, syntax score, complex coronary anatomy, diffuse coronary atherosclerosis. We restricted our searches to clinical trial, controlled clinical trial, dataset, multicenter study, observational study, pragmatic clinical trial, and randomized controlled trial. No restrictions were applied regarding language, sample size, or follow-up duration (see Supplementary material online, *Table S2*).

### Eligibility and study selection criteria

Publications were selected and reviewed by two cardiologist authors (N.C. and A.S.), with differences resolved by consensus. We included published studies that compared patients with CTO-PCI to patients without CTO-PCI, including those with OMT and/or those with failed CTO-PCI and OMT. If more than one study reported outcomes of the same cohort, we included the most recent or most comprehensive study. Studies reporting at least cardiovascular mortality were included (other reported outcomes are summarized in Supplementary material online, *Table S3*).

We excluded studies that did not report mean SS, had a mixed population (PCI and CABG treated CTO), or had no control group and follow-up time less than 9 months.

Patient and lesion characteristics were extracted on a per-protocol analysis, including follow-up time, age, sex, main cardiovascular risk factors, left ventricular ejection fraction (LVEF%), number of vessel diseases, mean SS, and number of CTO-PCI failures (see Supplementary material online, *Table S4*).

### Definition of study endpoints

Cardiovascular mortality per year was estimated as described in the statistical analysis section below, used as the primary endpoint, and subsequently correlated with SS. Secondary endpoints were myocardial infarction (MI), repeat revascularization, target vessel revascularization (TVR), cerebrovascular stroke, and major adverse cardiac events (MACE), defined as the composite of cardiovascular death, MI, and TLR. All the secondary outcomes were reported as estimated incidence per year, as the primary outcome.

Studies were also divided into low, intermediate, and high SS, using respectively ≤22, 23–32, ≥33 scores as cut-offs.^11^

The quality of included studies was independently assessed by two authors (N.C., A.S.) using GRADE classification (see Supplementary material online, *Table S5*). In addition, for each included study, we evaluated the quality of eligible studies using the Cochrane tool for risk of bias of nonrandomized studies of interventions, ROBINS-I (see Supplementary material online, *Table S6* and *Figure S1*), and RoB-2 for randomized trials (see Supplementary material online, *Table S7*).

### Statistical analysis

Continuous variables were reported as mean ± SD or median (IQR) for numeric data, while categorical variables were expressed as numbers and percentages. Statistical pooling for incidence estimates was performed using a fixed-effect or random-effect model, with generic inverse-variance weighting based on statistical homogeneity, and risk estimates with 95% confidence intervals (CIs) were computed using STATA/MP version 17.0 (STATA Corp, College Station, TX). Successful CTO-PCI and no-CTO-PCI groups were compared. The Cardiovascular mortality per year was calculated as −ln(1−observed cardiovascular mortality)/follow-up time (years). This value was subsequently used to derive the hazard ratio and corresponding standard errors for meta-analysis. Outcomes were analysed in randomized and observational trials, both separately and together. Results were reported as Hazard Ratio (HR) with 95% CIs. In addition, meta-regression analysis and subgroup analysis were performed to determine the influence of SS and baseline features on the primary outcome.

Hypothesis testing for statistical homogeneity was set at 2-tailed *P* < 0.05 and based on the Cochran Q test, with heterogeneity score (I^2^) values of 25%, 50%, and 75% representing mild, moderate, and severe heterogeneity, respectively.

## Results

### Included studies and patient demographics

**Figure 1:**
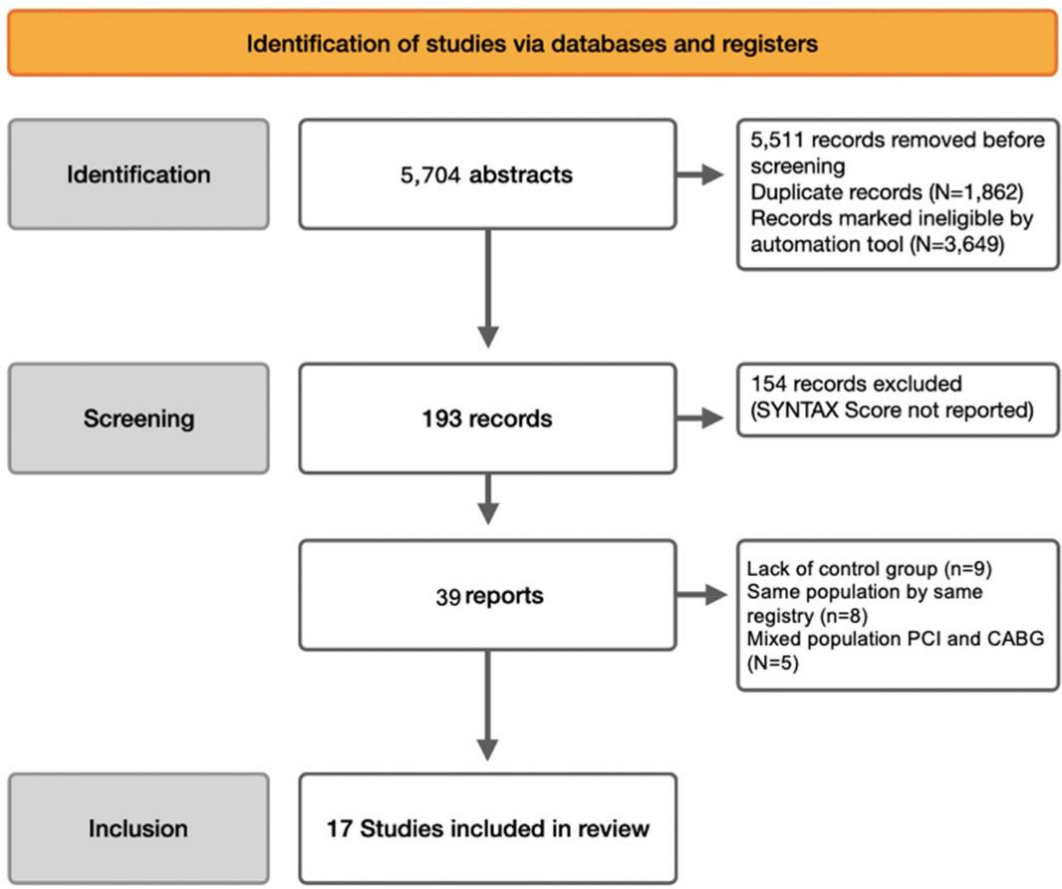
PRISMA 2020 flow diagram for new systematic reviews that included searches of databases and registers only. Flowchart summarizing study identification, screening, and inclusion for the meta-analysis.

The flowchart for the search strategy and study inclusion is presented in ***Figure 1***. From 193 abstracts, 154 were excluded due to a lack of SS, 8 due to data derived from the same population registry, 5 due to mixed population PCI and CABG, and 8 due to a lack of a control group. Finally, 17 studies^27–43^ were eligible for inclusion in this meta-analysis; 3 were prospective randomized trials, and 14 were prospective observational studies. The mean follow-up period was 4.2 (95%-CI 3.2–5.6) years.

Patient’s characteristics are summarized in ***Table 1***, a total of 11 001 patients with a mean age of 63.0 ± 10.5 years, of whom 8740 were male (80%), 7048 patients (74%) affected by MVD with a mean SS of 20.0 ± 8.2 (median 21.9, min 10.33, max 40.73, Q1 19.31, Q3 24.20), and mean LVEF% was 52.6 ± 10.0. The CTO-treated vessel was the left descending artery (LAD) in 36% of cases, the Circumflex artery (Cx) in 22%, and the Right coronary artery (RCA) in 48% of CTO-vessels. Baseline characteristics differed between groups, in particular in the non-CTO-PCI group, revealing a higher cardiovascular risk population as evidenced in the summarized ***Table 1***.

**Table 1:** Baseline characteristics of included cohorts

	Overall N = 11’001	CTO-PCI group N = 6’359	non-CTO-PCI group N = 4’642	Missing data in the N study/ N pz (%)	*P* value
Follow up (y) (IC 95%)	4,2 (3,2–5,6)				
Age (y)	63,0 ± 10,5	61,3 ± 10,4	64,4 ± 10,5	0	<0.001
Male	8’740 (81)	5’136 (80)	3'604 (80)	1/214 (1.9)	0.79
Hypertension	7'111 (66)	4'037 (63)	3'074 (67)	1/214 (1.9)	<0.001
Dyslipidemia	6'046 (57)	3'536 (58)	2'510 (57)	2/466 (4.2)	0.379
Diabetes	3'998 (36)	2'185 (34)	1'813 (39)	0	<0.001
Prev. MI	3’725 (36)	1'980 (34)	1'745 (40)	1/790 (7.1)	<0.001
Prev. PCI	1'971 (18)	1'211 (19)	760 (17.6)	1/402 (3.6)	0.068
LVEF % (mean)	52.6 ± 10.0	53.9 ± 9.8	50.75 ± 10.2	1/1072 (9.7)	<0.001
Multi vessel	7'048 (74)	3'811 (71)	3'237 (77)	3/1489 (13.5)	<0.001
CTO Vessel				2/1510 (13.7)	
LAD CTO	3'409 (36)	2'262 (40)	1'147 (29)		<0.001
Cx CTO	2'120 (22)	1'163 (20)	957 (24)		<0.001
RCA CTO	4'645 (48)	2'592 (46)	2'053 (53)		<0.001
SYNTAX (mean)	20.0 ± 8.2	19.5 ± 8.0	20.8 ± 8.5	0	<0.001

Summary of baseline patient characteristics, including demographics, coronary anatomy, and left ventricular function in the overall cohort and between study groups.

### Endpoint analysis

**Figure 2:**
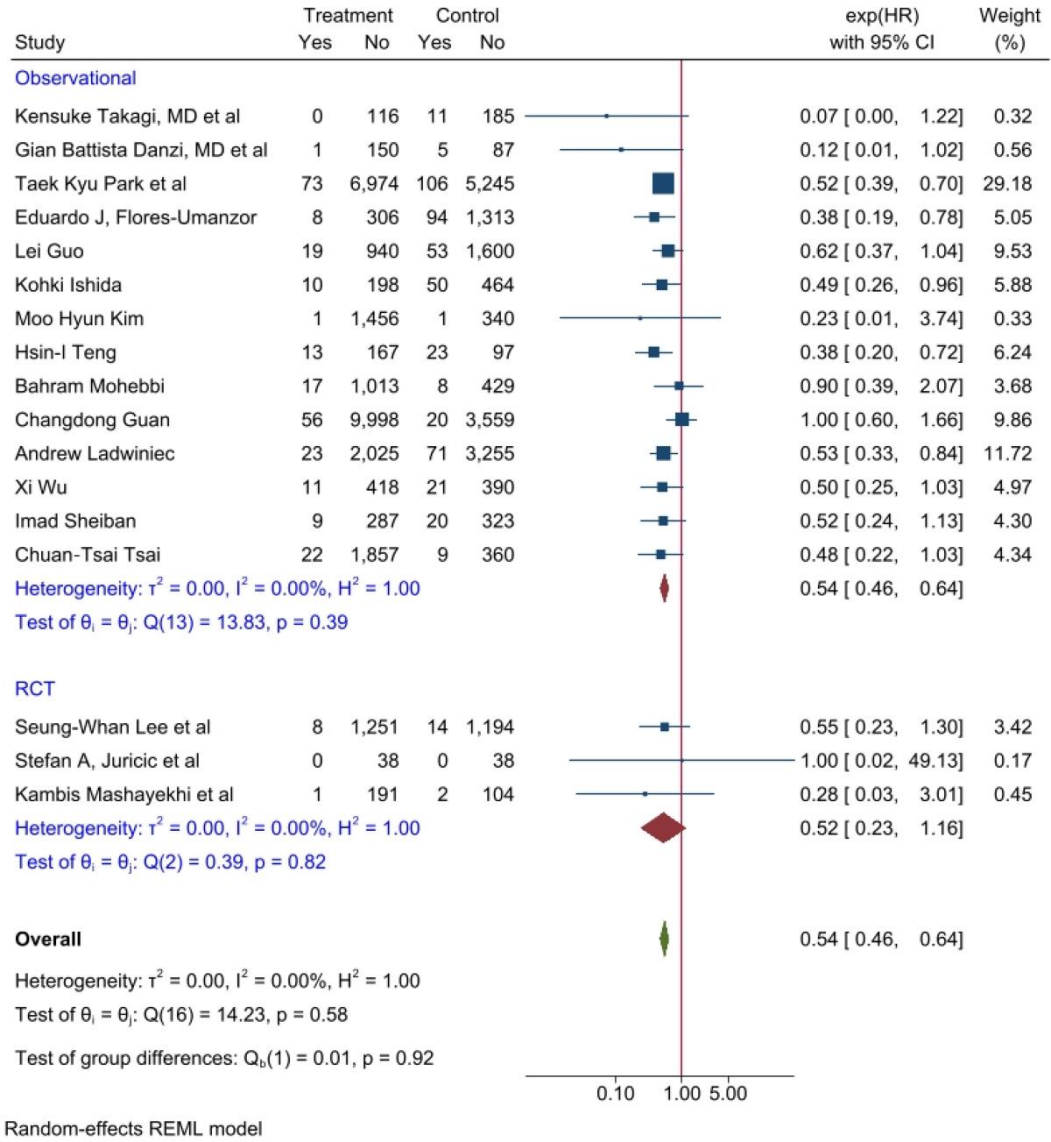
Cardiovascular mortality per year forrest plot of CTO-PCI vs. non-CTO-PCI, expressed as hazard ratio, sub-grouped by type of study (observational vs randomized trial studies). Forest plot illustrating the effect of CTO-PCI compared to non-CTO-PCI on cardiovascular mortality. Overall analysis showed a significant reduction in cardiovascular mortality (HR 0.54, 95% CI 0.46–0.64, *P* < 0.001), mainly driven by observational studies, while RCTs showed a non-significant trend (HR 0.52, 95% CI 0.23–1.15, *P* = 0.10).

Overall, cardiovascular mortality was lower with CTO-PCI than with non-CTO-PCI, with a Hazard ratio (HR) of 0.54 (95% CI 0.46–0.64, *P* < 0.001). The impact mainly resulted from trained observational studies, while in RCT trials, the subgroup resulted only in a trend with an HR of 0.52 but not statistically significant (95%-CI 0.23–1.15, *P* = 0.10) (***Figure 2***).

**Figure 3:**
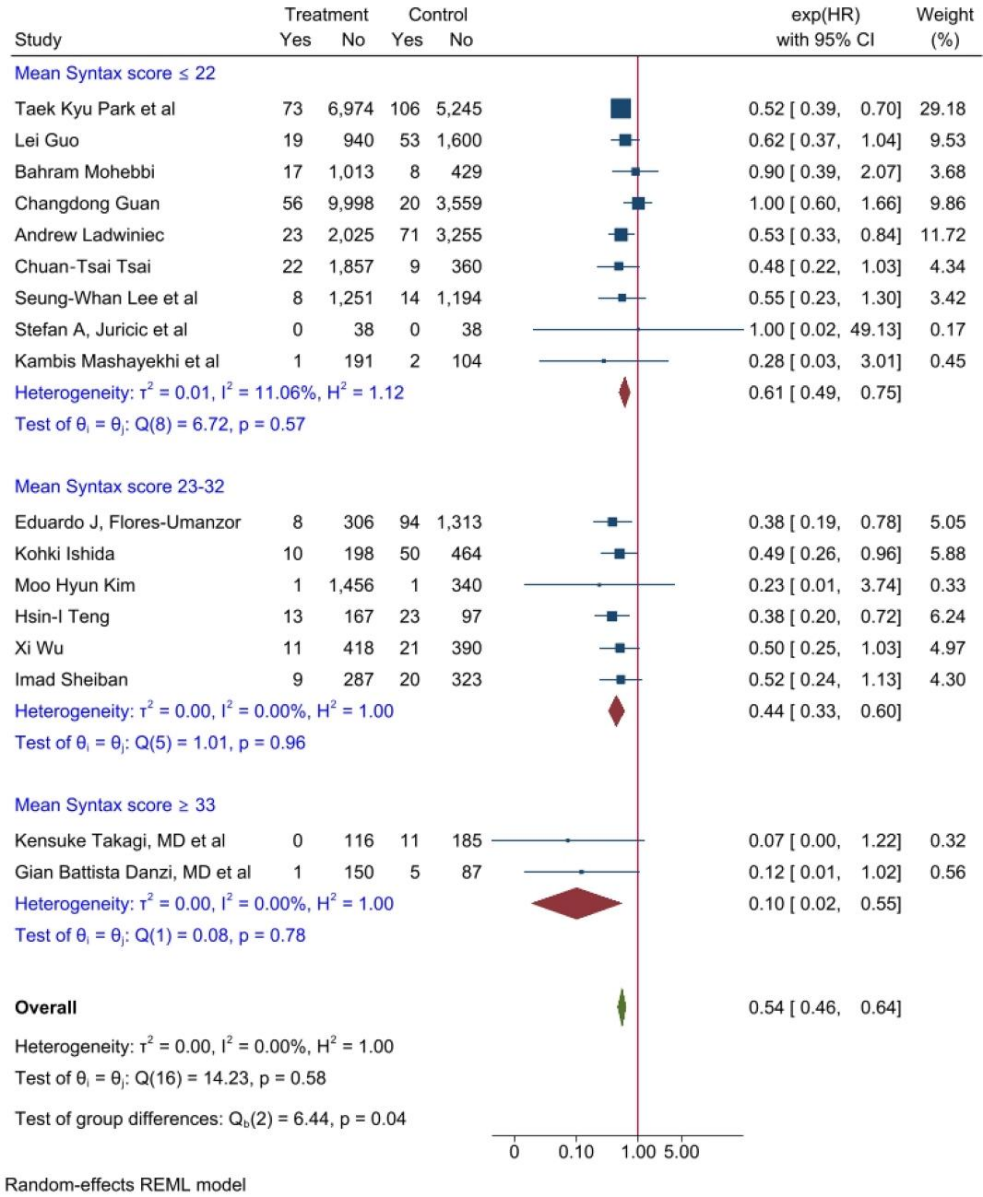
Cardiovascular mortality per year forrest plot of CTO-PCI vs. non-CTO-PCI, expressed as hazard ratio, sub-grouped by syntax score class risk (≤22, 23–32, ≥33). Forest plot illustrating the effect of CTO-PCI vs. non-CTO-PCI on cardiovascular mortality stratified by SYNTAX score (SS) risk. The benefit of CTO-PCI increased with higher SS categories (HR 0.61, 0.44, and 0.10 for low, intermediate, and high risk, respectively; *P* for trend = 0.04).

Studies were then further stratified according to their mean SS risk (low ≤22, intermediate 23–32, high≥33), and the magnitude of the effect was directly proportional to SS class risk. Specifically, HRs were 0.61, 0.44, and 0.10, respectively, in the low, intermediate, and high SS risk groups, and a *P*-value for trend of 0.04 was observed (***Figure 3***). The higher the complexity of the disease, expressed by the SS, the higher the effect size on HR by CTO-PCI.

**Figure 4:**
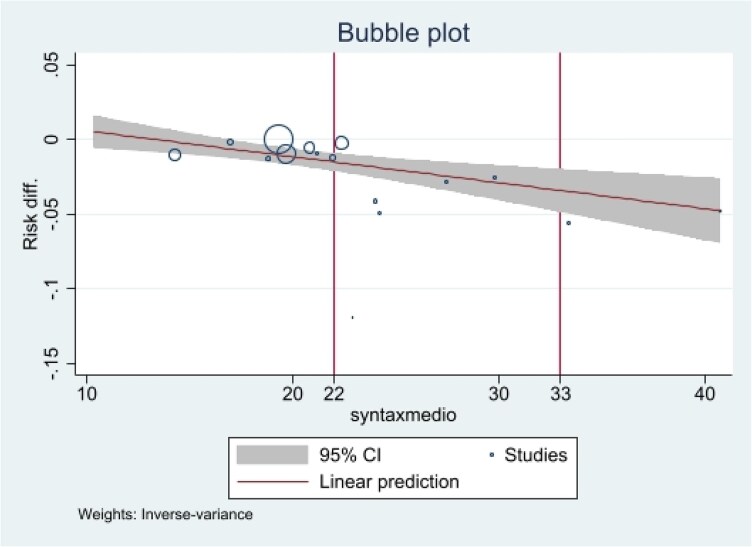
Meta-regression analysis of mean syntax score on cardiovascular mortality per year expressed as risk difference of CTO-PCI compared to non-CTO-PCI. Meta-regression showing the relationship between SYNTAX score (SS) and the cardiovascular mortality benefit of CTO-PCI vs. non-CTO-PCI. A positive linear correlation was observed, with an absolute risk reduction of 1.5% for every 10-point increase in SS (*P* = 0.005).

Furthermore, a positive linear correlation was observed between the benefit of CTO-PCI on cardiovascular mortality and SS using risk difference meta-regression analysis, with an absolute risk reduction of 1.5% per 10-point increase in SS (*P* = 0.005) (***Figure 4***).

### Secondary endpoints and subgroup analyses

Sub-group analysis demonstrated an association between the CTO-PCI effect on cardiovascular mortality per year and LVEF and age. Specifically, HR was 0.49 (95%-CI 0.37–0.64, *P* < 0.001) in studies with a mean LVEF ≤50% compared to HR of 0.63 (95%-CI 0.46–0.85, *P* = 0.003) in the mean LVEF ≥50% group, although without reaching statistical significance difference between groups (*P* = 0.23) (see Supplementary material online, *Figure S2*). Moreover, by sub-grouping studies by mean age from 60 to >70 years, with increases of 5 years per sub-group, a trend in HR reduction was observed: 0.97 vs. 0.53 vs. 0.49 vs. 0.38 (*P* for trend 0.04) (see Supplementary material online, *Figure S3*).

Regarding secondary outcomes, no effect was observed in any revascularization (HR 0.93, 95%-CI 0.69–1.28, *P* = 0.68), cerebrovascular stroke (HR 0.63, 95%-CI 0.35–1.16, *P* = 0.14), or MI (HR 1.27, 95%-CI 0.83–1.95, *P* = 0.27). There was a higher percentage of TLR in the CTO-PCI group with an HR of 1.42 (95%-CI 1.02–1.99, *P* = 0.04) in observational studies, while no effect was observed in RCT. Ultimately, CTO-PCI had a positive effect on MACE with an HR of 0.77 (95%-CI 0.66–0.92, *P* = 0.004) in observational studies, despite the result on TLR. (see Supplementary material online, *Table S5*).

### Sub-analysis

Demographic, clinical, and coronary disease mean characteristics between the observational study and the randomized trial are reported in ***Table 2*.** Intrestingly, in observational studies there were higher percentage of patients with diabetes (36% vs. 32%, *P* = 0.006), previous MI (38% vs. 20%, *P* < 0.001), lower mean LVEF% (45 ± 10 vs. 56 ± 10, *P* < 0.001) and higher mean SS (24 ± 9 vs. 15 ± 6, *P* < 0.001), compared to randomized trials. These differences could explain the different effect of CTO-PCI on cardiovascular mortality per year observed between observational and randomized studies.

**Table 2:** Demographic, clinical and coronary disease mean characteristics between the observational study and randomized trial.

Variable	Overall (n 17)	Observational (n 14)	RCT (n 3)	*P* value
Number of patients (total)	11 001	9881	1120	
Years	3,2 ± 1,7	3,6 ± 1,6	1,6 ± 1,2	<0.001
Number of PCI	6359 (57%)	5791 (58%)	568 (51%)	<0.001
Age	65 ± 10	65 ± 10	64 ± 8	<0.001
Male	8740/10 893 (80%)	7814/9773 (80%)	926 (83%)	0.030
Diabetes Mellitus	3998 (36%)	3637 (36%)	361 (32%)	0.006
Hypertension	7111/10 897 (65%)	6351/9867 (64%)	760 (68%)	0.02
Dyslipidemia	6046/10 641 (57%)	5509/9726 (57%)	537/915 (59%)	0.23
Previous PCI	1971/10 615 (18%)	1771/9585 (18%)	200 (18%)	0.61
Previous MI	3725/10 227 (36%)	3505/9197 (38%)	220 (20%)	<0.001
LVEF%	47 ± 10	45 ± 10	56 ± 10	<0.001
Syntax score	22 ± 8	24 ± 9	15 ± 6	<0.001
MVD	7048/9528 (74%)	6277/8498 (74%)	771 (69%)	<0.001
LAD CTO	3409/9507 (35%)	3004/8477 (35%)	405 (36%)	0.63

Demographic, clinical, and coronary disease characteristics in observational studies and randomized trials. Observational studies included a higher prevalence of diabetes and previous MI, lower mean LVEF%, and higher mean SYNTAX score compared with randomized trials.

### Publication bias

Assessment of potential publication bias did not reveal significant evidence: the Galbraith plot did not show relevant deviations and Egger’s test was not statistically significant (β = −0.54, SE = 0.49, *P* = 0.27); however, visual inspection of the funnel plot suggests a slight asymmetry, with a possible lack of small studies not supporting CTO-PCI (see Supplementary material online, *Figure S4A* and *B*).

## Discussion

In this meta-analysis, successful CTO-PCI in patients with MVD was associated with a reduction of cardiovascular mortality. Importantly, the benefit was amplified by the extent and complexity of the atherosclerotic disease burden, as reflected by the SS.

While our study population consisted mainly of patients with preserved left ventricular ejection fraction (LVEF), signals emerged suggesting a potential additional benefit in those with impaired LVEF and MVD.

No significant effects were observed for myocardial infarction (MI), target lesion revascularization (TLR), overall revascularization, or cerebrovascular stroke. It should be noted, however, that TLR outcomes may have been confounded by reinterventions on non-CTO vessels. Furthermore, in contrast to the favourable results from observational studies, randomized controlled trials (RCTs) did not demonstrate a mortality benefit for CTO-PCI, underscoring the complexity of translating registry data into trial settings.^26,44^

Current guidelines reflect this uncertainty. The 2018 European guidelines classify CTO-PCI as a Class IIa indication,^11^ whereas the 2021 American guidelines assign it a Class IIb recommendation.^10^ Both, however, strongly advocate for complete revascularization in MVD (Class I)^11^ without further addressing how to deal with CTO lesions. Given that MVD is present in the majority of CTO patients (52–87%),^26^ our findings may help clarify this apparent discrepancy: outcomes might depend less on the CTO itself and more on the overall codominant extent of non-occluded coronary disease. This is important because a greater extent of non-occluded disease increases the risk of donor vessel destabilization, which may elevate the risk of sudden death. Supporting this, Goel *et al*. demonstrated that long-term outcomes after CTO-PCI are determined not only by procedural success but also by whether the CTO-PCI procedure contributes to achieving complete revascularization.^45^ Similarly, Valenti *et al.*^16^ reported that the survival benefit of CTO-PCI is closely tied to the global burden of CAD rather than to CTO recanalisation alone. Evidence from the SYNTAX trial and subsequent studies confirms that the mortality benefit is most significant in patients with high SS (≥33) and in those with diffuse MVD^19,46–48^

These data raise the possibility that the disease burden in non-occluded arteries primarily mediates the prognostic advantage of CTO-PCI. Indeed, Valenti *et al*.^16^ showed that patients with an isolated CTO and no significant non-CTO disease had very low mortality regardless of whether the CTO was recanalized. Conversely, in MVD, successful CTO-PCI emerged as the strongest independent predictor of survival.^46,47,49^ Aslan *et al*.^18^ further identified MVD with high SS as an independent predictor of all-cause mortality, reinforcing this observation.

This interpretation is also supported by studies such as Shiba *et al*., who demonstrated that successful CTO-PCI improved outcomes in patients with high SS but not in those with low SS.^46^ Taken together, these findings indicate that the SS, as an integrated measure of overall coronary complexity, is a key surrogate for the clinical benefit of CTO-PCI.

Pathophysiological mechanisms may provide additional insight. Revascularization of a CTO may protect against catastrophic events involving donor vessels by restoring antegrade flow and enabling reverse collateralisation, functionally resembling a bypass graft. Unrevascularized CTOs have been linked to a threefold higher risk of sudden cardiac death (SCD),^21^ primarily driven by refractory cardiac arrest that remains unresponsive to resuscitation.^3,4,6,22^ Supporting this, Yannopoulos *et al*. and Kosmopoulos *et al*. found strong associations between CTOs, complex CAD, and refractory OHCA.^3,4^ Registry data further reveal particularly poor outcomes in patients with CTO and ventricular tachyarrhythmias or cardiogenic shock, especially in those with high SS or multiple CTOs.^23,24^

Anatomical studies strengthen this concept. Fujimoto *et al*.^25^ showed that patients with a CTO in a donor or non-culprit vessel had higher mortality than those with a critical (99%) stenosis in the same vessel. Shinouchi *et al*.^25^ provided an electrophysiological perspective, reporting that ACS patients with concurrent CTOs were more likely to present with pulseless electrical activity (PEA) arrest—an arrhythmia unresponsive to defibrillation.

These observations suggest that the prognostic relevance of CTO-PCI extends beyond ischaemia relief. While ischaemia reduction remains an important therapeutic goal, non-invasive stress testing has proven insufficient to predict survival benefit in the CTO setting.^9,50,51^ Instead, emerging data from coronary computed tomography angiography (CCTA) highlight the global atherosclerotic burden as the dominant determinant of prognosis, independent of ischaemia.^7,17^ This may represent a conceptual shift: rather than focusing solely on ischaemia, patient selection for CTO-PCI should consider the overall extent of CAD and MVD.

In summary, our meta-analysis suggests that the mortality benefit of CTO-PCI arises primarily in patients with high global atherosclerotic burden and MVD. The SS effectively captures this risk and may help refine patient selection. This pathophysiological insight underscores the importance of viewing CTO-PCI not only as a means of ischaemia relief but also as a potential strategy to mitigate the catastrophic outcomes associated with complex CAD and donor-vessel destabilization.

## Conclusion

Overall, CAD burden seems to influence how CTO-PCI relates to cardiovascular mortality. The strongest survival benefit is seen in patients with high SYNTAX scores, indicating a move from ischaemia-based choices to decisions based on anatomical details. These findings apply to a PCI-selected population, as CABG-treated patients were not included. Future randomized controlled trials should focus on high-SS patients to confirm these results.

## Limitations

Our meta-analysis included both randomized and non-randomized trials. Although RCTs represent the gold standard in clinical research, their number in this field remains limited, and the intermediate SYNTAX scores reported in RCTs were relatively low. Consequently, the predominance of observational studies constitutes an important limitation. Nevertheless, heterogeneity across studies was low (I^2^ = 0%, *P* = 0.58) (*Figure 2*). An additional limitation of our meta-analysis is that studies that did not report SYNTAX Scores were excluded. Furthermore, only two studies included patients with a SYNTAX Score >33, and these patients were deemed ineligible for surgical revascularization. It is therefore extremely challenging to identify additional studies in this high SYNTAX population, and this remains an inherent limitation of our analysis. As such, our findings may still be influenced by confounding variables, including age and left ventricular ejection fraction (LVEF), as highlighted in the subgroup analyses.

Another limitation is that the non-CTO-PCI comparator group included patients who had failed CTO-PCI. Only 11 studies (3767 patients) provided data on failed procedures, with 997 failures (36.2%). This factor may have significantly impacted the overall outcomes. However, meta-regression by percentage of failure included in each study did not show any association, and the results remained statistically significant in a sensitivity analysis excluding observational studies with a higher prevalence of failure CTO-PCI in the non-CTO PCI group (OR 0.50; 95% CI 0.41–0.614; *P* < 0.0001) (see Supplementary material online, *Figures S5* and *S6*).

Finally, the possibility of publication bias must be acknowledged. Funnel plot asymmetry suggested potential bias, although Egger’s test did not reach statistical significance (see Supplementary material online, *Figure S4A* and *B*). Small studies with neutral or negative findings on the mortality benefit of CTO-PCI are less likely to be published, potentially skewing the evidence base.

## Supplementary Material

oeag045_Supplementary_Data

## Data Availability

The data underlying this article are available in the published studies included in this meta-analysis and in the article and its supplementary material.
