# Supplementary material for: Overall coronary disease burden modifies the prognostic benefit of CTO-PCI: a SYNTAX score–stratified meta-analysis
Source: Eur Heart J Open. 2026 Mar 12;6(2):oeag045. doi: 10.1093/ehjopen/oeag045 (PMC13042282; doi:10.1093/ehjopen/oeag045)

**Table S1. PRISMA meta-analysis checklist of items to include when reporting a systematic review involving a meta-analysis check page number**

| **Section and Topic** | **Item #** | **Checklist item** | **Location where item is reported** |
| --- | --- | --- | --- |
| **TITLE** | | |  |
| Title | 1 | Identify the report as a systematic review:  *The Prognostic Benefit of CTO-PCI and the Complexity of Coronary Artery Disease: A systematic review and meta analysis* | 1 |
| **ABSTRACT** | | |  |
| Abstract | 2 | See the PRISMA 2020 for Abstracts checklist. | 2-3 |
| **INTRODUCTION** | | |  |
| Rationale | 3 | Describe the rationale for the review in the context of existing knowledge.  *CTO-PCI randomized trials don’t show any prognostic impact compared to CTO-OMT, while data from observational trials suggest the opposite. This may be explained by the presence of lower cardiovascular risk/complex CAD patients in trials than observational studies.* | 4-5 |
| Objectives | 4 | Provide an explicit statement of the objective(s) or question(s) the review addresses.  *Does the prognostic impact of coronary chronic total occlusion revascularization depend on the complexity of coronary disease? (PICO)* | 5 |
| **METHODS** | | |  |
| Eligibility criteria | 5 | Specify the inclusion and exclusion criteria for the review and how studies were grouped for the syntheses.  *Studies that evaluated the mortality in patients underwent successful chronic total occlusion percutaneous revascularization (CTO-PCI) vs. patient not revascularized (CTO-no-PCI), and that reported the Syntax Score (SS) of the cohorts of the patients were included. Studies were excluded if cardiovascular mortality data was not reported.* | 6-7-8 |
| Information sources | 6 | Specify all databases, registers, websites, organisations, reference lists and other sources searched or consulted to identify studies. Specify the date when each source was last searched or consulted.  *A systematic review was conducted through PubMed, MEDLINE, Embase, Google Scholar and Cochrane databases until April 2024* | 6-7-8 |
| Search strategy | 7 | Present the full search strategies for all databases, registers and websites, including any filters and limits used.  *Reported Table S2* | 6-7-8 |
| Selection process | 8 | Specify the methods used to decide whether a study met the inclusion criteria of the review, including how many reviewers screened each record and each report retrieved, whether they worked independently, and if applicable, details of automation tools used in the process.  *All studies were independently reviewed and selected by two interventional cardiologists.* | 6-7-8 |
| Data collection process | 9 | Specify the methods used to collect data from reports, including how many reviewers collected data from each report, whether they worked independently, any processes for obtaining or confirming data from study investigators, and if applicable, details of automation tools used in the process.  *Data were extracted independently and in duplicate by 2 investigators and summarized by a third investigator* | 6-7-8 |
| Data items | 10a | List and define all outcomes for which data were sought. Specify whether all results that were compatible with each outcome domain in each study were sought (e.g. for all measures, time points, analyses), and if not, the methods used to decide which results to collect.  All cause death, Cardiovascular death, Non fatal Myocardial infarction, Stroke/TIA, Any revascularization, TLR defined as PCI on CTO vessel, MACE defined as Cardiovascular death/any revascularization/Non fatal MI. | 6-7-8 |
|  | 10b | List and define all other variables for which data were sought (e.g. participant and intervention characteristics, funding sources). Describe any assumptions made about any missing or unclear information.  *Follow up days mean, age, sex, previous PCI, previous MI, previous Stroke, Diabetes, Hypertension, Dyslipidemia, LVEF; SYNTAX, Multi vessel disease, CTO vessel, failure PCI, Complete revascularization.* | 6-7-8 |
| Study risk of bias assessment | 11 | Specify the methods used to assess risk of bias in the included studies, including details of the tool(s) used, how many reviewers assessed each study and whether they worked independently, and if applicable, details of automation tools used in the process.  *Risk of bias were independently assessed by two interventional cardiologists using ROBINS-I tool for Observational studies and RoB 2 for RCT* | 6-7-8 |
| Effect measures | 12 | Specify for each outcome the effect measure(s) (e.g. risk ratio, mean difference) used in the synthesis or presentation of results.  *Risk difference* | 6-7-8 |
| Synthesis methods | 13a | Describe the processes used to decide which studies were eligible for each synthesis (e.g. tabulating the study intervention characteristics and comparing against the planned groups for each synthesis (item #5)).  *Each study was evaluated tabulating the CTO-PCI group characteristics and comparing against CTO-OMT/failed PCI group for each synthesis* | 6-7-8 |
|  | 13b | Describe any methods required to prepare the data for presentation or synthesis, such as handling of missing summary statistics, or data conversions: *Number of multi vessel disease patients were calculated by adding 2 vessel disease and 3 vessel disease. LVEF was considered reduced if mean was inferior to 50%.* | 6-7-8 |
|  | 13c | Describe any methods used to tabulate or visually display results of individual studies and syntheses.  *Data were summarized on tables and forrest plot* | 6-7-8 |
|  | 13d | Describe any methods used to synthesize results and provide a rationale for the choice(s). If meta-analysis was performed, describe the model(s), method(s) to identify the presence and extent of statistical heterogeneity, and software package(s) used.  *Random-effect meta analysis using restricted maximum likelihood (REML) estimation for risk difference. Heterogeneity was assessed by I^2^ heterogeneity test. Statistical analysis was performed with STATA 17 MP.* | 6-7-8 |
|  | 13e | Describe any methods used to explore possible causes of heterogeneity among study results (e.g. subgroup analysis, meta-regression).  *After observing other variable differences between group, subgroup analysis was performed for categorical variables and meta-regression for continuous variables.* | 6-7-8 |
|  | 13f | Describe any sensitivity analyses conducted to assess robustness of the synthesized results.  *None* |  |
| Reporting bias assessment | 14 | Describe any methods used to assess risk of bias due to missing results in a synthesis (arising from reporting biases).  *None search for unpublished data* |  |
| Certainty assessment | 15 | Describe any methods used to assess certainty (or confidence) in the body of evidence for an outcome.  GRADE scale | 6-7-8 |
| **RESULTS** | | |  |
| Study selection | 16a | Describe the results of the search and selection process, from the number of records identified in the search to the number of studies included in the review, ideally using a flow diagram. *Figure S1* | 9 |
|  | 16b | Cite studies that might appear to meet the inclusion criteria, but which were excluded, and explain why they were excluded.  *None* |  |
| Study characteristics | 17 | Cite each included study and present its characteristics. | *Table S4* |
| Risk of bias in studies | 18 | Present assessments of risk of bias for each included study. | *Table S6-S7 Figure S1* |
| Results of individual studies | 19 | For all outcomes, present, for each study: (a) summary statistics for each group (where appropriate) and (b) an effect estimate and its precision (e.g. confidence/credible interval), ideally using structured tables or plots.  *Figure 1 (Forrest plot)* | Table S5 |
| Results of syntheses | 20a | For each synthesis, briefly summarize the characteristics and risk of bias among contributing studies. | Table S5-s6 |
|  | 20b | Present results of all statistical syntheses conducted. If meta-analysis was done, present for each the summary estimate and its precision (e.g. confidence/credible interval) and measures of statistical heterogeneity. If comparing groups, describe the direction of the effect. | 9-10-11 |
|  | 20c | Present results of all investigations of possible causes of heterogeneity among study results. Sub analisys and meta-regression figures | 9-10-11 |
|  | 20d | Present results of all sensitivity analyses conducted to assess the robustness of the synthesized results. *None* |  |
| Reporting biases | 21 | Present assessments of risk of bias due to missing results (arising from reporting biases) for each synthesis assessed. *Funel plot* | Figure S4 |
| Certainty of evidence | 22 | Present assessments of certainty (or confidence) in the body of evidence for each outcome assessed. *GRADE* | Table S5 |
| **DISCUSSION** | | |  |
| Discussion | 23a | Provide a general interpretation of the results in the context of other evidence. | 12-13-14 |
|  | 23b | Discuss any limitations of the evidence included in the review. | 12-13-14 |
|  | 23c | Discuss any limitations of the review processes used. | 12-13-14 |
|  | 23d | Discuss implications of the results for practice, policy, and future research. | 12-13-14 |
| **OTHER INFORMATION** | | |  |
| Registration and protocol | 24a | Provide registration information for the review, including register name and registration number, or state that the review was not registered.  *PROSPERO CRD42023477306* | 6 |
|  | 24b | Indicate where the review protocol can be accessed, or state that a protocol was not prepared. | 6 |
|  | 24c | Describe and explain any amendments to information provided at registration or in the protocol. | 6 |
| Support | 25 | Describe sources of financial or non-financial support for the review, and the role of the funders or sponsors in the review. | 6 |
| Competing interests | 26 | Declare any competing interests of review authors. | 6 |
| Availability of data, code and other materials | 27 | Report which of the following are publicly available and where they can be found: template data collection forms; data extracted from included studies; data used for all analyses; analytic code; any other materials used in the review. *Supplementary* | 6 |

PICOS = population, intervention, comparators, outcomes, study design.

Abbreviations: PCI: Percutaneous coronary intervention; CTO: Chronic totally occluded; OMT: Optimal medical therapy; CAD: Coronary artery disease; MACE: Major adverse cardiac events; TLR: target lesion revascularization; TIA: Transient ischemic attack; MI: Myocardial infarction; LVEF: Left ventricular ejection fraction;

**Table S2. Representative search string on PubMed**

| 1 | (((((((((((((((((((("CTO"[All Fields]) OR ("chronic occluded coronary"[All Fields])) OR ("chronic occluded coronary arteries"[All Fields])) OR ("chronic totally occluded coronary"[All Fields])) OR ("occluded coronary"[All Fields])) AND ("percutaneous coronary intervention"[All Fields]))) OR ("pci"[All Fields])) OR ("coronary intervention"[All Fields])) OR ("angioplasty"[All Fields])) OR ("coronary angioplasty"[All Fields])) AND (mortality)) OR ("death"[All Fields])) OR ("cardiac death"[All Fields])) OR ("cardiac mortality"[All Fields])) OR ("all cause death"[All Fields])) OR ("cardiovascular death"[All Fields])) AND ("syntax"[All Fields])) OR ("syntax score"[All Fields])) OR ("complex coronary anatomy"[All Fields])) OR ("diffuse coronary atherosclerosis"[All Fields]) | 5,511 |
| --- | --- | --- |
| 2 | Filters: Clinical Trial; Controlled Clinical Trial; Dataset; Multicenter Study; Observational Study; Pragmatic Clinical Trial; Randomized Controlled Trial | 193 |
| *A similar search strategy was employed for MEDLINE, Embase, Google Scholar and Cochrane databases | | |

**Table S3. Outcome data available from each study**

| **Study** | **All-cause Death** | **Cardiovascular Death** | **MI** | **MACE** | **Repeat revascularization** | **TLR** | **Stroke/**  **TIA** |
| --- | --- | --- | --- | --- | --- | --- | --- |
| Kensuke, 2013 | Yes | Yes | No | No | No | Yes | No |
| Danzi, 2013 | Yes | Yes | Yes | No | Yes | Yes | Yes |
| Lee, 2019 | Yes | Yes | Yes | Yes | Yes | Yes | Yes |
| Park, 2021 | Yes | Yes | Yes | No | No | No | No |
| Juricic, 2021 | Yes | Yes | Yes | No | Yes | No | No |
| Mashayekhi, 2018 | Yes | Yes | Yes | Yes | Yes | Yes | No |
| Flores-Umanzor, 2019 | Yes | Yes | Yes | No | Yes | No | No |
| Guo, 2020 | Yes | Yes | Yes | Yes | No | Yes | No |
| Ishida, 2020 | Yes | Yes | Yes | Yes | No | No | No |
| Kim, 2016 | No | Yes | Yes | Yes | No | No | Yes |
| Teng, 2018 | No | Yes | Yes | Yes | No | Yes | No |
| Mohebbi, 2023 | Yes | Yes | Yes | Yes | No | Yes | Yes |
| Ladwiniec, 2015 | Yes | Yes | No | No | No | No | No |
| Guan, 2021 | Yes | Yes | Yes | No | Yes | Yes | No |
| Wu, 2022 | Yes | Yes | Yes | Yes | Yes | Yes | Yes |
| Sheiban, 2022 | Yes | Yes | Yes | No | No | Yes | Yes |
| Tsai, 2020 | Yes | Yes | Yes | Yes | No | No | No |

**Table S4. Baseline characteristics of included studies.**

| **Author** | **Study design** | **N** | **Mean Age*** | **Follow up (days)** | **MVD** | **LVEF% mean** | **Prev. PCI** | **Prev. MI** | **SYNTAX score mean** | **Entry criteria** | **Outcomes** | **N failure PCI** |
| --- | --- | --- | --- | --- | --- | --- | --- | --- | --- | --- | --- | --- |
| Kensuke, 2013 | Registry | 29 / 46 | 63 / 65 | 1466 | 17 (58)/ 26 (56) | 51,7/47,8 | 21 (72)/19 (41) | 18 (62)/25 (54) | 33,3/33,4 | consecutive patients with ULM stenosis treated with DES. RCA CTO-PCI vs RCA CTO OMT/failure | All cause death, C.V. death, TLR | 7 failure |
| Danzi, 2013 | Registry | 76/44 | 66/70 | 365 | 41 (54)/28 (64) | 42/38 | 21 (28)/19 (43) | 18 (23)/25 (57) | 40/42 | consecutive patients who underwent PCI for at least 2 CTOs from Florence CTO registry | All cause death, C.V. death, TLR, Any revasc, MI, stroke | 44 pz (34 uncomplete, 10 failure) |
| Lee, 2019 | RCT | 417/398 | 62/63 | 1095 | 302(72)/288(72) | 57,3/57,6 | 64(15)/75(19) | 45(11)/34(8) | 20,8/20,8 | Pz with silent ischemia, stable angina or acute coronary syndrome (DECISION TRIAL) | All cause death, C.V. death, TLR, Any revasc, MI, stroke, MACE*, MACCE | 29 PCI, 78 No-PCI crossover  41 Failure |
| Park, 2021 | Registry | 883/664 | 61/66 | 2883 | 595(67)/517(78) | 57,6/53,6 | 178(20)/208(31) | 180(20)/211(32) | 19,6/19;8 | CTO who had symptomatic angina and/or a positive functional ischemia study.  prior coronary artery bypass graft, ST‐segment–elevation myocardial infarction, or cardiogenic shock or cardiopulmonary resuscitation as initial presentation were excluded | All cause death, C.V. death, MI | N.R. |
| Juricic, 2021 | RCT | 50/50 | 61/63 | 275 | 0/0 | 54,9/51,3 | 29(58)/35(70) | 0/0 | 10,8/9,9 | objective proof of myocardial ischemia and/or proof of myocardial viability in akinetic regions. exclusion criteria were an angiographically significant stenosis in the non-CTO coronary artery, MI in the previous 90 days, LVEF 25%, other severe clinical conditions | All cause death, C.V. death, MI (secondary endpoint) | 3 failure |
| Mashayekhi, 2018 | RCT | 191/104 | 65/68 | 365 | 87(86)/94(90) | 54,7/59,6 | 39 (38)/38 (36) | 28 (28)/33 (32) | 14/16 | CTO-PCI eligible patients with symptoms, CMR. Exclusion criteria were LVEF <25%, acute coronary syndromes within 72 h preceding the index procedure, and contraindications to cMRI | All cause death, C.V. death, MI, Any revasc., TLR (secondary endpoint) | 4 failure |
| Flores-Umanzor, 2019 | Registry | 76/326 | 67/70 | 1470 | 62(82)/281(86) | 46,9/43 | N.R. | 21(28)/108(33) | 22,7/24,3 | DM patients with CTO. | All cause death, C.V. death, MI, Any revasc., | N.R. |
| Guo, 2020 | Registry | 470/800 | 63/65 | 730 | 332(71)/707(88) | 54,5/51,7 | 63(13)/78(10) | 183(39)/406(51) | 19,6/23,3 | previous coronary artery bypass grafting (CABG) or developed acute ST-segment elevation myocardial infarction during the preceding 48 h were excluded | C.V. Death, MI, TVR, MACE** | 264 failure |
| Ishida, 2020 | Registry | 64/150 | 62/67 | 1129 | 54(84)/107(71) | 29,2/30,5 | 8(12)/22(15) | 32(50)/50(70) | 24,7/24 | Patients with at least one CTO and reduced LVEF. | All cause Death, C.V. death, MI, MACE*** | 18 failure |
| Kim, 2016 | Registry | 355/83 | 61/62 | 1497 | N.R. | 50,4/50,8 | 55(15)/9(11) | 112(31)/20(24) | 22,6/21,4 | Consecutive CTO patients, excluding incomplete or terminated PCI | C.V. Death, MI, Stroke MACE° | 83 failure |
| Teng, 2018 | Registry | 67/39 | 81/75 | 912 | 63(94)/38(97) | 44/30 | 19/(28)/4(10) | 12 (18)/5 (13) | 20,5/27 | NSTEMI patients with CTO. | C.V. Death, MI, TLR, MACE°° | 28 failure |
| Mohebbi, 2023 | Registry | 555/235 | 59/60 | 666 | N.R. | 43/41 | 145(26)/55(23) | N.R | 16,4/18,4 | All patients with CTO | C.V. Death, Stroke, MI, TLR, MACE°°° | 235 failure |
| Guan, 2021 | Registry | 1961/698 | 57/57 | 1861 | 1454(74)/578(83) | 60,5/60,4 | 178(9)/69(10) | 852(43)/257(37) | 19,2/19,7 | All CTO-PCI. Suboptimal recanalization group was excluded from our analysis | C.V. death MI (primary endpoint), All cause Death, Any revasc., TLR, TVR. | 698 failure |
| Ladwiniec, 2015 | Registry | 405/651 | 63/66 | 1825 | 212(52)/388(60) | N.R. | 22(5)/64(10) | 202(50)/394(61) | 14,5/14,5 | CTO patients, excluded those treated for acute MI in the territory of CTO in the preceding three months, prior CABG, mitral or aortic valve disease of moderate severity or greater, active neoplastic. Excluded non-dominant right coronary artery | All cause death, C.V. Death, C.V. death/MI, Any revasc. | 155 failure (considered in PCI group) |
| Wu, 2022 | Registry | 135/126 | 65/69 | 1131 | N.R. | 30,9/31,4 | 27(20)/8(6) | 73(54)/40(32) | 29,3/30,3 | CTO and LVEF  ≤  40%. angina, and/or myocardium viability in the territory of CTO | MACCE§, All cause death, C.V. death, TVR, IM, Stroke | 34 failure |
| Sheiban, 2022 | Registry | 96/108 | 68/67 | 1090 | 91/(95)/79(73) | 43/42 | 13(13)/28(26) | 60(62/62(57) | 28/27 | unprotected LMCA, who underwent PCI and have CTO | All cause death, CV. Death, MI, TLR, Stroke | 22 failure |
| Tsai, 2020 | Registry | 619/120 | 67/69 | 1095 | 501(81)/106(88) | 49/48 | 317(51)/66(55) | 135 (22)/24(20) | 18,6/20 | CTO patients with angina or evidence of ischemia. Patients with acute coronary syndrome and end stage renal disease on renal replacement therapy were excluded | All cause death, C.V. Death, MI, MACE§§ | 120 failure |

* MACE: Cardiovascular death, MI, stroke, or any revascularization; **MACE: Cardiovascular death, MI, TVR.

***MACE: Cardiovascular death, MI; °MACE: Cardiovascular death, MI, Stroke;

°°MACE: Cardiovascular death, MI, TLR; °°°MACE: Cardiovasculare death, MI, TLR, Stroke/TIA

§MACCE: all-cause death (ACD), cardiac mortality, relapse of myocardium infarction, targeted lesion angiogenesis, rehospitalisation, cardiac failure, and stroke.

§§MACE: All cause death, Cardiovascular death, MI.

**Supplementary table S5: quality of included studies assessed by GRADE classification**

| **Outcomes** | **Anticipated absolute effects^*^** (95% CI) | | Relative effect (95% CI) | № of participants (studies) | Certainty of the evidence (GRADE) |
| --- | --- | --- | --- | --- | --- |
|  | **Risk with no-CTO-PCI** | **Risk with CTO-PCI** |  |  |  |
| **Cardiovascular Mortality per Year** | 2.8% | **1.5%**  (1.3 to 1.8%) | **HR 0.54** (0.46 to 0.64) | 43552 (14 non-randomised studies) | ⨁⨁⨁◯ Moderate^a^ |
| **Cardiovascular Mortality per Year** | 1.2% | **0.6%** (0.3 to 1.4%) | **HR 0.52** (0.23 to 1.16) | 2724 (3 RCTs) | ⨁⨁⨁⨁ High |
| **All cause Mortality per Year** | 4.8% | **2.8%** (2.2 to 3.5%) | **HR 0.58** (0.46 to 0.73) | 37509 (10 non-randomised studies) | ⨁⨁⨁◯ Moderate |
| **All cause Mortality per Year** | 1.7% | **1.2%** (0.6 to 1.3%) | **HR 0.68** (0.36 to 0.73) | 2724 (3 RCTs) | ⨁⨁⨁⨁ High |
| **MI per Year** | 1.7% | **1.5%** (1.1 to 2.1%) | **HR 0.91** (0.66 to 1.24) | 37308 (11 non-randomised studies) | ⨁⨁◯◯ Low |
| **MI per Year** | 2.6% | **3.3%** (2.2 to 5.0%) | **HR 1.27** (0.83 to 1.95) | 2724 (3 RCTs) | ⨁⨁⨁⨁ High |
| **Any Revascularization per Year** | 4.6% | **4.5%** (3.2 to 6.4%) | **HR 0.99** (0.70 to 1.42) | 35451 (6 non-randomised studies) | ⨁⨁◯◯ Low |
| **Any Revascularization per Year** | 4.3% | **3.0%** (1.2 to 7.5%) | **HR 0.69** (0.27 to 1.75) | 2724 (3 RCTs) | ⨁⨁⨁⨁ High |
| **TLR per Year** | 0.8% | **1.2%** (0.9 to 1.7%) | **HR 1.42** (1.02 to 1.99) | 29438 (8 non-randomised studies) | ⨁⨁◯◯ Low |
| **TLR per Year** | 3.3% | **2.0%** (0.6 to 6.5%) | **HR 0.61** (0.18 to 2.01) | 2724 (3 RCTs) | ⨁⨁⨁⨁ High |
| **Stroke/TIA per year** | 0.8% | **0.5%** (0.2 to 1.2%) | **HR 0.66** (0.30 to 1.43) | 4891 (5 non-randomised studies) | ⨁⨁◯◯ Low |
| **Stroke/TIA per year** | 0.8% | **0.5%** (0.2 to 1.3%) | **HR 0.60** (0.22 to 1.58) | 2519 (2 RCTs) | ⨁⨁⨁⨁ High |
| **MACE per Year (CV death + MI + TLR)** | 7.9% | **6.2%** (5.2 to 7.3%) | **HR 0.77** (0.65 to 0.92) | 43552 (14 non-randomised studies) | ⨁⨁⨁◯ Moderate |
| **MACE per Year (CV death + MI + TLR)** | 8.0% | **6.0%** (2.6 to 13.5%) | **HR 0.74** (0.32 to 1.73) | 2724 (3 RCTs) | ⨁⨁⨁⨁ High |

1. ***The risk in the intervention group** (and its 95% confidence interval) is based on the assumed risk in the comparison group and the **relative effect** of the intervention (and its 95% CI).
   **CI:** confidence interval; **HR:** hazard ratio
2. **GRADE Working Group grades of evidence**
   **High certainty:** we are very confident that the true effect lies close to that of the estimate of the effect.
   **Moderate certainty:** we are moderately confident in the effect estimate: the true effect is likely to be close to the estimate of the effect, but there is a possibility that it is substantially different.
   **Low certainty:** our confidence in the effect estimate is limited: the true effect may be substantially different from the estimate of the effect.
   **Very low certainty:** we have very little confidence in the effect estimate: the true effect is likely to be substantially different from the estimate of effect.

**Table S6: Risk of Bias assessment observational studies with ROBINS-I tool**

| **Author** | **Confounding** | **Selection of participants into the study** | **Classification of interventions** | **Deviations from intended interventions** | **Missing data** | **Mesaurement of the outcome** | **Selection of the reported result** |
| --- | --- | --- | --- | --- | --- | --- | --- |
| **Kensuke, 2013** | **Serious risk**  High syntax patients were treated with CABG unless High surgical risk. High complex CTO probably were treated with OMT. Failure PCI were considered in OMT group | **Moderate risk**  Consecutive patients | **Moderate risk**  PCI CTO vs CTO OMT-failure PCI | **Moderate risk** | **Not Reported** | **Moderate risk**  assessors are aware of intervention status | **Low risk**  Primary end point analysis |
| **Danzi, 2013** | **Serious risk**  As prev. studies | **Moderate risk**  As previous | **Moderate risk**  As previous | **Moderate risk** | **Low risk** 0% | **Moderate risk**  As previous | **Low risk**  As previous |
| **Park, 2021** | **Serious risk**  As prev. studies | **Moderate risk**  As previous | **Moderate risk**  As previous | **Moderate risk** | **N.R.** | **Moderate risk**  As previous | **Low risk**  As previous |
| **Flores-Umanzor, 2019** | **Moderate risk**  As prev. studies but propensity matched | **Moderate risk**  As previous | **Moderate risk**  As previous | **Moderate risk** | **N.R.** | **Moderate risk**  As previous | **Low risk**  As previous |
| **Guo, 2020** | **Serious risk**  As prev. studies | **Moderate risk**  As previous | **Moderate risk**  As previous | **Moderate risk** | **N.R.** | **Moderate risk**  As previous | **Low risk**  As previous |
| **Ishida, 2020** | **Serious risk**  As prev. studies | **Moderate risk**  As previous | **Moderate risk**  As previous | **Moderate risk** | **N.R.** | **Moderate risk**  As previous | **Low risk**  As previous |
| **Kim, 2016** | **Serious risk**  As prev. studies | **Moderate risk**  As previous | **Moderate risk**  As previous | **Moderate risk** | **N.R.** | **Moderate risk**  As previous | **Low risk**  As previous |
| **Teng, 2018** | **Serious risk**  As prev. studies | **Moderate risk**  As previous | **Moderate risk**  As previous | **Moderate risk** | **N.R.** | **Moderate risk**  As previous | **Low risk**  As previous |
| **Mohebbi, 2023** | **Serious risk**  As prev. studies | **Moderate risk**  As previous | **Moderate risk**  As previous | **Moderate risk** | **Low Risk**  0% | **Moderate risk**  As previous | **Low risk**  As previous |
| **Guan, 2021** | **Serious risk**  As prev. studies | **Moderate risk**  As previous | **Moderate risk**  As previous | **Moderate risk** | **Moderate Risk**  9,5% | **Moderate risk**  As previous | **Low risk**  As previous |
| **Ladwiniec, 2015** | **Serious risk**  As prev. studies | **Moderate risk**  As previous | **Critical risk**  39% of PCI group were failure | **Moderate risk** | **N.R.** | **Low risk**  Report confirmed by clinical audit officer. | **Low risk**  As previous |
| **Wu, 2022** | **Serious risk**  As prev. studies | **Moderate risk**  As previous | **Moderate risk**  As previous | **Moderate risk** | **N.R.** | **Moderate risk**  As previous | **Low risk**  As previous |
| **Sheiban, 2022** | **Serious risk**  As prev. studies | **Moderate risk**  As previous | **Moderate risk**  As previous | **Moderate risk** | **N.R.** | **Moderate risk**  As previous | **Low risk**  As previous |
| **Tsai, 2020** | **Serious risk**  As prev. studies | **Moderate risk**  As previous | **Moderate risk**  As previous | **Moderate risk** | **N.R.** | **Moderate risk**  As previous | **Low risk**  As previous |

N.B. The most studies were designed and conducted similarly

**Figure S1: Plot and summary risk of bias assessment with ROBINS-I tool**

**
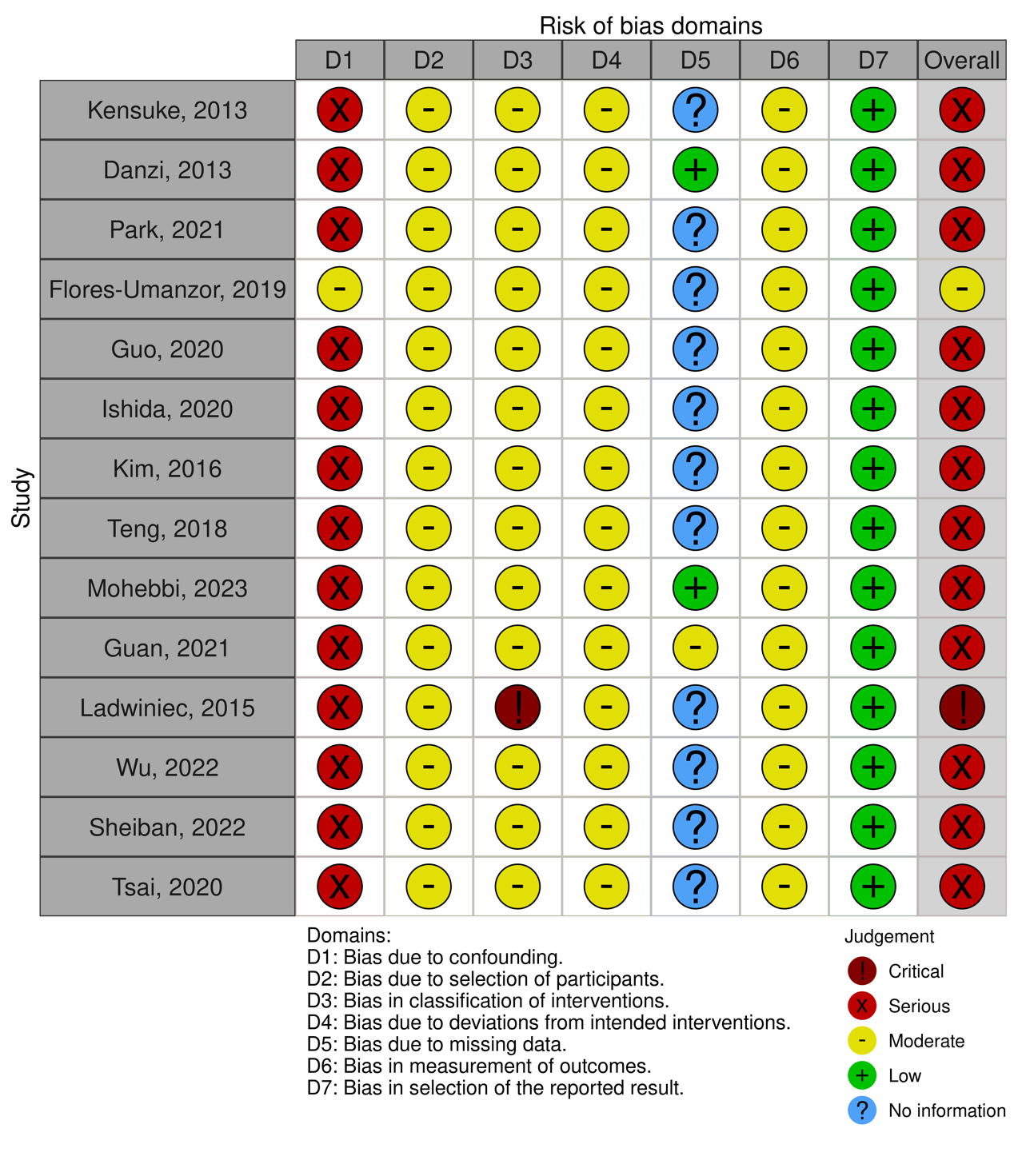
**

**
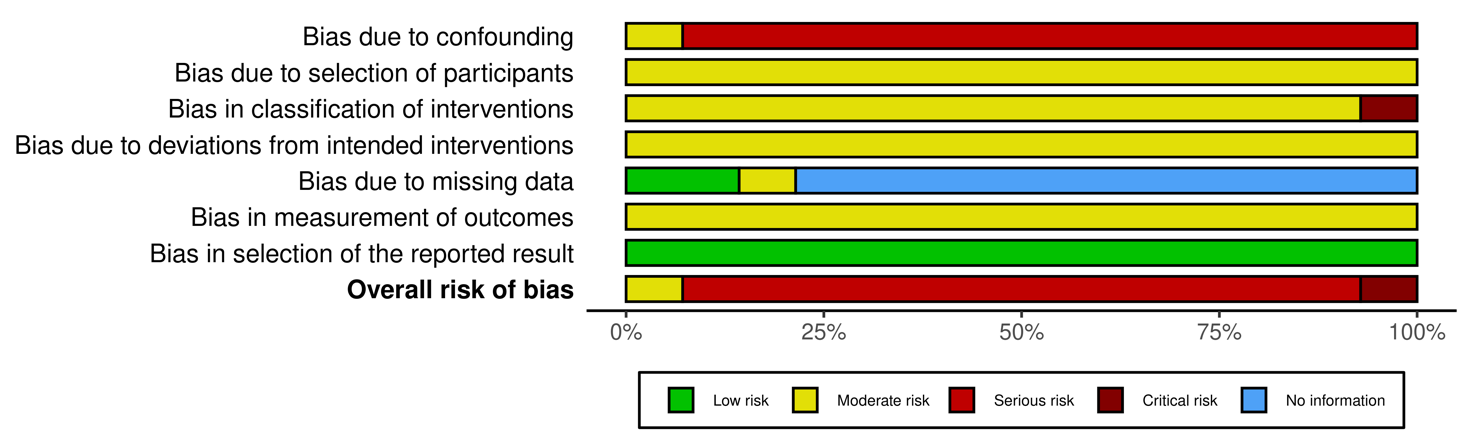
**

**Table S7: Risk of Bias assessment RCT with RoB 2 tool**

| **Author** | **Random Sequence Generation** | **Allocation Concealment** | **Blinding of Participants and Personnel** | **Blinding of Outcome Assessment** | **Incomplete Outcome Data** | **Selective Reporting** | **Other Bias** |
| --- | --- | --- | --- | --- | --- | --- | --- |
| **Lee, 2019** | **Low risk** Computer-generated system | **Low risk** Computer-generated system | **High risk**  Open label | **Moderate risk**  Clinical events committee is not blinded | **Low risk**  Minimal missing data (10%) | **Low risk**  pre-specified outcomes reported | **Unclear**  Source of funding not stated |
| **Juricic, 2021** | **Low risk** Computer-generated system | **Low risk** Computer-generated system | **High risk**  Open label | **Moderate risk**  Clinical events committee is not blinded | **Low risk**  Minimal missing data (1 pz) | **Moderate risk**  Secondary outcomes | **Unclear**  Source of funding not stated |
| **Mashayekhi, 2018** | **Low risk** Computer-generated system | **Low risk** Computer-generated system | **High risk**  Open label | **Moderate risk**  Clinical events committee is not blinded | **Low risk**  Minimal missing data | **Moderate risk**  Secondary outcomes | **Unclear**  Source of funding not stated |

**Figure S2: Forest plot sub-grouped by LVEF 50% of CTO-PCI vs non-CTO-PCI effect on Cardiovascular mortality per year.**

**
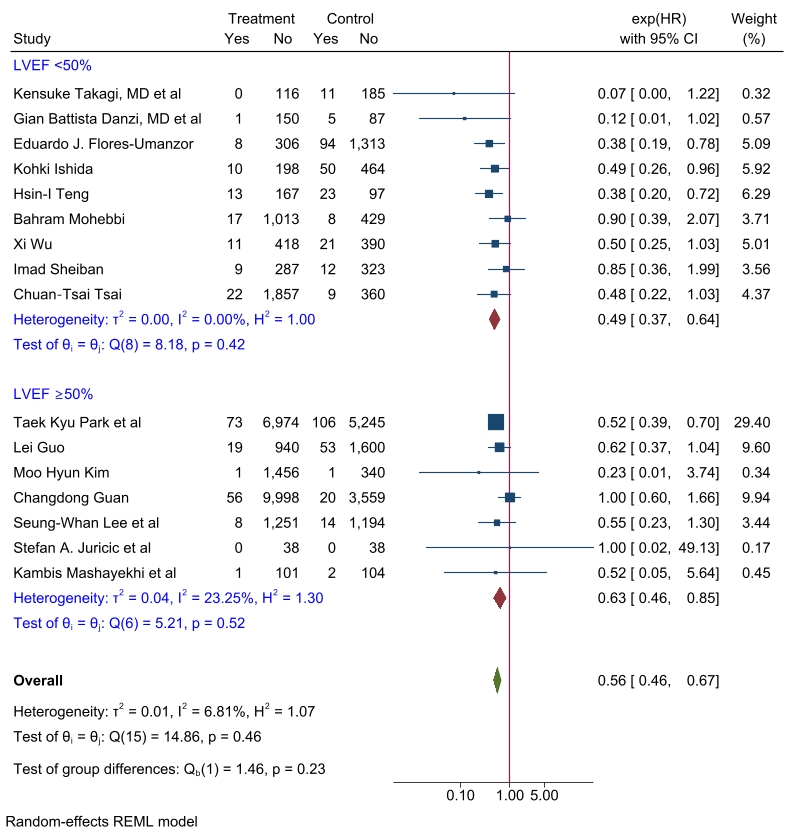
**

**Figure S3: Forest plot sub-grouped by age of CTO-PCI vs non-CTO-PCI effect on Cardiovascular mortality per year.**

**
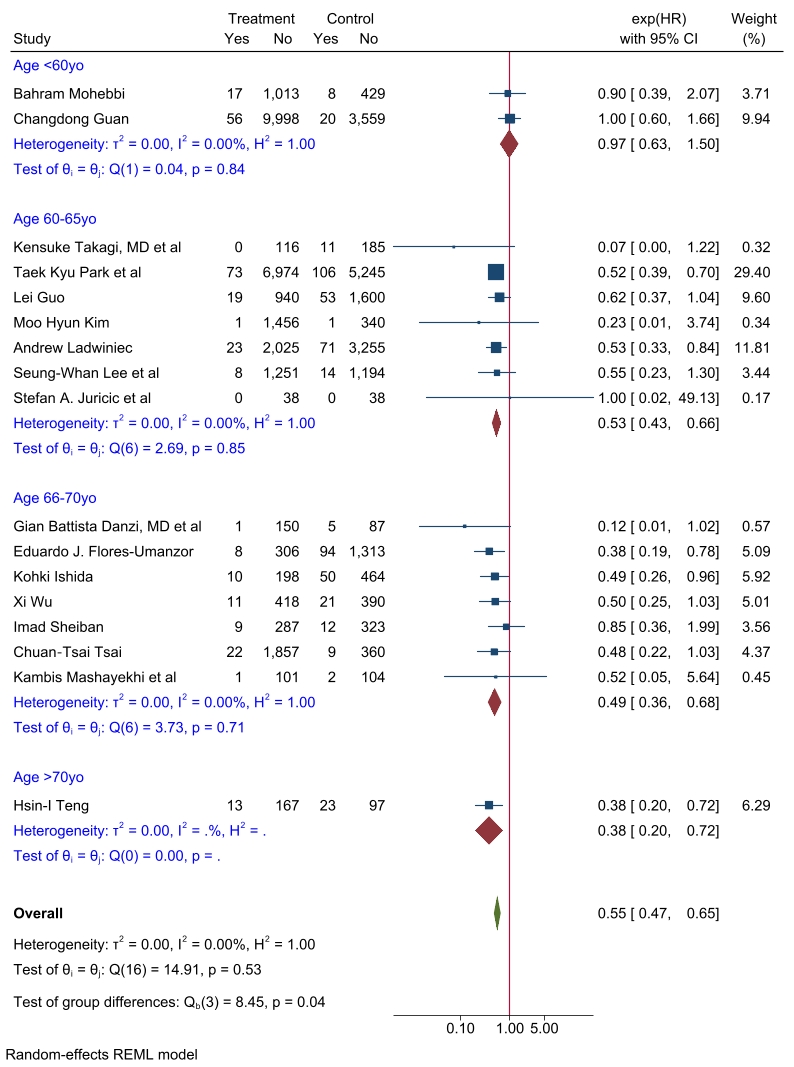
**

**Figure S4: Assessment of publication bias: Galbraith plot (A), funnel plot (B) evaluating small-study effects in the comparison between CTO-PCI and non-CTO PCI on annual cardiovascular mortality.**

**A**


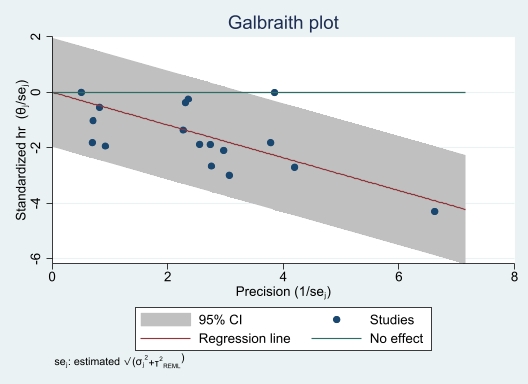


**B**


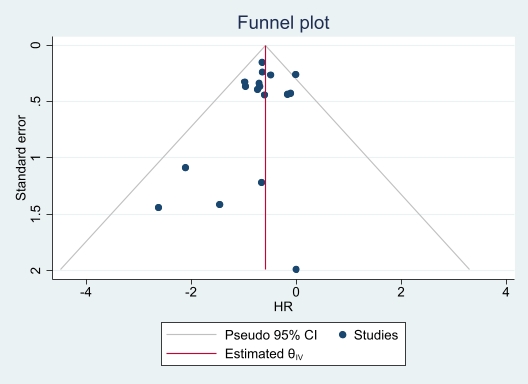


**Figure S5:**


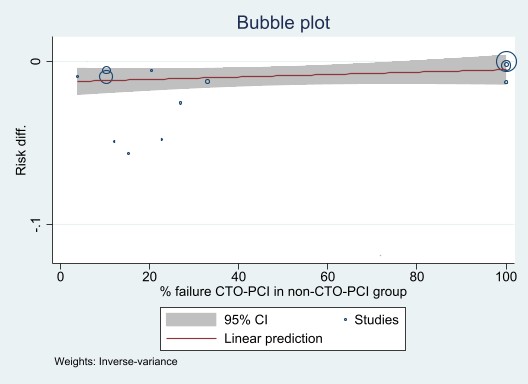


**Coefficient 0.0000743 (95% C.I. -0.0000617;0.0002103), p value 0.284**

**Figure S6:**


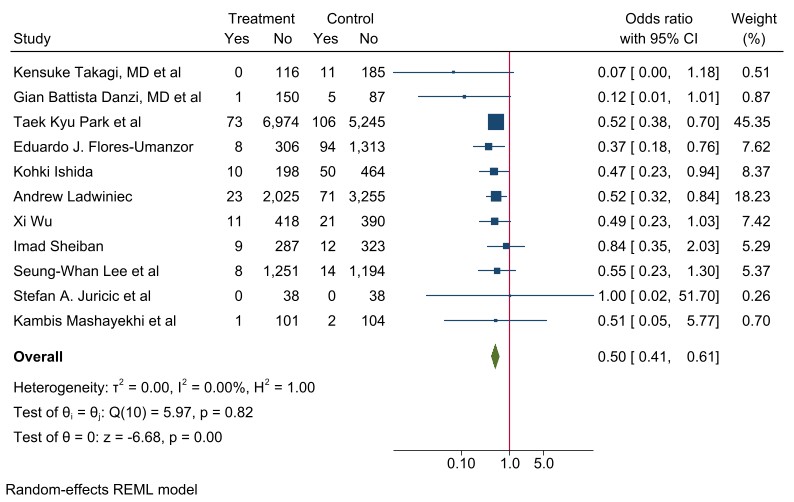

Supplement: oeag045_Supplementary_Data [file oeag045_supplementary_data.docx]
